# Effects of Polypropylene Microplastics and Copper Contamination on Rice Seedling Growth

**DOI:** 10.3390/nano16030196

**Published:** 2026-02-02

**Authors:** Ziwen Hao, Steven Xu, Siquan Huang, Lin Wang

**Affiliations:** 1 Miami College, Jinming Campus, Henan University, Kaifeng 475004, China; hzww@henu.edu.cn (Z.H.); 19228972029@163.com (S.H.); 2 Thermo Fischer Scientific-PPD Bioanalytical Laboratory, 3230 Deming Way, Middleton, WI 53563-1475, USA; scx1@scarletmail.rutgers.edu; 3 College of Geographical Sciences, Faculty of Geographical Science and Engineering, Henan University, Zhengzhou 450046, China

**Keywords:** polypropylene, microplastics, heavy metals, Cu^2+^, rice seedlings

## Abstract

This study investigates the effects of polypropylene microplastics (PP-MPs) and copper (Cu), applied individually and in combination, on the growth (root and shoot length, fresh and dry biomass), peroxidase (POD) activity, and Cu accumulation of rice seedlings. A hydroponic experiment was conducted with four treatments: control (CK), PP, Cu, and PP+Cu. Exposure to PP-MPs slightly promoted seedling growth, whereas Cu markedly inhibited growth and induced chlorosis. Compared with Cu alone, co-exposure to PP-MPs and Cu (PP+Cu) partially improved shoot growth and alleviated Cu-induced suppression of shoot POD activity. In contrast, root POD activity showed the strongest reduction under PP+Cu (91.7% decrease), revealing a pronounced root–shoot divergence in antioxidant responses. Moreover, total Cu accumulation in seedlings increased by 12.3% in PP+Cu relative to Cu alone, implying that PP-MPs may influence Cu bioavailability and/or internal partitioning. However, Cu speciation and subcellular distribution were not quantified in this study and should be examined in future work. Overall, PP-MPs may simultaneously enhance Cu uptake while partially mitigating shoot-level toxicity, underscoring the complexity of microplastic–metal co-contamination in rice seedling systems.

## 1. Introduction

The ubiquitous application of plastic materials across diverse industrial sectors is primarily driven by their exceptional performance-to-weight ratio, remarkable resilience, and significant cost-effectiveness. However, approximately 79% of waste plastics are ultimately destined for landfills or discarded in the natural environment, with only 9% being recycled [1,2]. The accumulation of plastic waste presents a severe and persistent danger to natural ecosystems [3]. Due to factors such as ultraviolet radiation, thermal oxidation, acid rain leaching, and mechanical grinding, discarded plastics in soil gradually break down into micronsized or even nanosized plastic particles [4]. Microplastics (MPs) are defined as plastic and fiber fragments with diameters smaller than 5 mm and are widely distributed in environmental systems [5]. As the volume of raw plastic waste continues to increase annually, microplastic pollution in terrestrial ecosystems has become an increasingly prominent issue and may represent one of the most widespread and persistent anthropogenic changes on Earth’s surface [6,7,8]. The global annual production of plastics is steadily rising, and if current production and waste management trends continue, it is estimated that by 2050, the world will generate around 798.58 million tons of plastic waste, with close to half (397.82 million tons) ending up in landfill sites [9]. Microplastics enter soils through various sources, including landfills, soil amendments, sewage sludge application, wastewater irrigation, compost and organic fertilizers, agricultural plastic film residues, tire wear, and atmospheric deposition. Microplastics are generated in terrestrial environments as soil-dwelling organisms mechanically and biologically degrade plastic debris through processes including ingestion, metabolic breakdown, and subsequent egestion [10].

Polypropylene microplastics (PP-MPs) are small plastic particles that have garnered attention due to their widespread distribution in the environment. The impact of microplastics on plant growth is bimodal, exhibiting either beneficial or detrimental outcomes contingent upon specific variables such as MP characteristics, concentration levels, duration of exposure, and the plant genotype [11]. For plants, MPs adhere to seed coats, root hairs, and cell wall pores, causing physical obstructions that inhibit seed germination, root elongation, and hinder water and nutrient uptake. MPs can also be absorbed by the roots through water absorption and intracellular ingestion, ultimately suppressing plant growth [12]. Jiang et al. found that MPs can accumulate in the roots of broad beans, blocking root pores and reducing water absorption, which delays seed germination [13,14]. Dong et al. demonstrated that MPs particles inhibit photosynthesis in rice by blocking stomata, reducing net photosynthetic rate, chlorophyll fluorescence, and chlorophyll a content [15]. Jiang et al. also showed that PP-MPs significantly reduced chlorophyll content in soybean leaves [16]. Plants play a key role in the soil ecosystem, contributing to material cycling, energy flow, and information transfer, and are also a source of food for humans [17]. When MPs are absorbed by plants in the soil, they not only affect normal growth and development but can also be transferred through the food chain to humans, posing significant health risks [18].

The high surface-area-to-volume ratio and hydrophobic nature of MPs facilitate the adsorption of diverse pollutants such as heavy metals, antibiotics, and persistent organic pollutants, thereby increasing their potential environmental mobility [19]. Due to their high adsorption capacity and persistence, MPs serve as carriers for heavy metals in soil–plant systems, altering the bioavailability and toxicity of pollutants [20]. Heavy metal pollution is a significant environmental issue facing the modern world. Rapid industrialization has led to the expansion of manufacturing, and improper disposal of industrial waste has resulted in increasing metal concentrations and severe pollution [21]. Once heavy metals enter the soil, they not only reduce crop yields and damage ecosystems but also enter the food web, threatening public well-being [22]. Cu exists naturally in soils as an essential trace metal, with an average concentration of about 30–35 mg·kg^−1^ and is found in many minerals [23]. The main sources of Cu pollution are mining, refining, waste incineration, and agricultural activities [24]. Cu functions as both a vital micronutrient and a potential toxin in plants, depending on its concentration, making it an ideal candidate to study the nuanced interactions between pollutants and plant physiological pathways. Cu serves as an essential cofactor for numerous enzymes and plays pivotal roles in regulating a wide array of physiological and metabolic pathways in plants. However, elevated concentrations of Cu can induce changes in DNA, cell membrane integrity, respiration, photosynthesis, and enzyme activity, leading to reduced growth and photosynthetic performance and ultimately threatening plant survival [25,26,27].

Beyond growth inhibition, microplastics in agroecosystems raise broader concerns because plants can act as an entry point for contaminants into the terrestrial food chain. Increasing evidence suggests that micro-/nanoplastics can be taken up by crop roots and translocated to aboveground tissues under certain particle sizes and exposure conditions, implying potential movement toward edible plant parts and possible trophic transfer. Such processes have been documented and summarized for crop systems including rice, and comprehensive reviews further highlight the potential implications of microplastic contamination for terrestrial food chains and food safety [28,29,30].

The extent of the effects caused by PP-MPs and Cu on soil health and plant development varies significantly, highlighting their complex ecological interactions. As a major staple crop cultivated worldwide, rice represents a suitable model plant for research into microplastic and heavy metal pollution. Therefore, this study further investigates the individual and combined impacts of Cu and PP-MPs on rice seedlings under hydroponic conditions.

## 2. Materials and Methods

### 2.1. Experimental Design

Rice seedlings (Guangxingyou 1380) were obtained from Hainan Shennong Gene Technology Co., Ltd. (Haikou, China). Polypropylene microplastics (PP-MPs; supplier-reported nominal diameter: 15 μm) were purchased from Kexinda Polymer Materials Co., Ltd. (Dongguan, China). Copper was supplied as analytical-grade copper sulfate pentahydrate (CuSO_4_·5H_2_O).

The experiment included four treatments: (1) control (CK, Hoagland nutrient solution), (2) PP (Hoagland solution + PP-MPs at 50 mg·L^−1^), (3) Cu (Hoagland solution + Cu^2+^ at 20 mg·L^−1^), and (4) PP+Cu (Hoagland solution containing PP-MPs at 50 mg·L^−1^ and Cu^2+^ at 20 mg·L^−1^). Each treatment comprised five independent flasks (biological replicates; n = 5), and each flask contained 3 rice seedlings. These seedlings whose size and physiological status were visually consistent, and randomly assigned them to treatment flasks. The flask was treated as the experimental unit.

A hydroponic system was established using 500 mL Erlenmeyer flasks. Three healthy seedlings with similar size and physiological status were placed in each flask, and 150 mL of exposure medium was added. Seedling roots were fully immersed, and flask openings were covered with aluminum foil to reduce contamination and evaporation.

All flasks were incubated in a controlled-environment chamber (BRS-WHM-2000G, Ningbo Pulante Instrument Co., Ltd., Ningbo, China) at 25 °C under a 14 h light/10 h dark photoperiod for 7 days. Evaporative and transpirational water loss was replenished daily using a micropipette with deionized water to maintain a constant volume and minimize unintended concentration changes in the exposure media.

After 7 days, root length, shoot length, fresh and dry biomass, peroxidase (POD) activity, and Cu concentration in seedlings were determined.

### 2.2. Preparation of Solutions

Fe–EDTA stock solution was prepared by weighing 3.73 g EDTA–Na_2_ and 2.78 g FeSO_4_·4H_2_O. Deionized water was boiled and cooled to ~70 °C. EDTA–Na_2_ was dissolved in 300 mL of the cooled water with continuous stirring until completely dissolved. FeSO_4_·4H_2_O was dissolved separately in 200 mL of the cooled water. The FeSO_4_ solution was then slowly added into the EDTA solution under continuous stirring to form the Fe–EDTA complex. The resulting 500 mL stock solution was transferred to an amber glass bottle and stored in the dark at cool temperature until use.

The Hoagland nutrient solution was prepared following Jin et al. [31]. The macronutrients per 1 L were: 1.18 g Ca(NO_3_)_2_·4H_2_O, 0.51 g KNO_3_, 0.49 g MgSO_4_·7H_2_O, and 0.14 g KH_2_PO_4_. The micronutrients per 1 L were: 2.86 mg H_3_BO_3_, 1.81 mg MnCl_2_·4H_2_O, 0.22 mg ZnSO_4_·7H_2_O, 0.08 mg CuSO_4_·5H_2_O, and 0.02 mg H_2_FMnO_4_·H_2_O. All reagents were weighed using an analytical balance. Each salt was first dissolved separately in a small volume of distilled water and then combined. Subsequently, 2 mL of Fe-EDTA solution was added, and the mixture was brought to a final volume of 1 L with distilled water.

Preparation of Cu^2+^ solution: Cu^2+^ exposure medium (20 mg·L^−1^ as Cu^2+^) was prepared by dissolving 0.0786 g of CuSO_4_·5H_2_O in distilled water, followed by thorough stirring until fully dissolved, and then adjusting the final volume to 1.0 L with distilled water.

Preparation of PP-MP suspension: A 50 mg·L^−1^ PP-MP suspension was prepared by adding 0.0500 g of polypropylene microplastic powder to the Hoagland nutrient solution and bringing the total volume to 1.0 L. The suspension was sonicated for 30 min using an ultrasonic homogenizer (400 W, 20.5 kHz, Xinzhi JY98-IIIN, Ningbo, China) to improve dispersion.

Preparation of combined PP+Cu medium: For the combined treatment, 0.0500 g of PP-MPs was first dispersed in a small volume of Hoagland nutrient solution by sonication in a water bath for 30 min. The previously prepared Cu^2+^ solution was then added, and the mixture was brought to a final volume of 1.0 L with Hoagland nutrient solution to obtain the PP–Cu composite exposure medium. The final concentrations in the combined medium were 50 mg·L^−1^ PP-MPs and 20 mg·L^−1^ Cu^2+^, as the solution was adjusted to the final volume after mixing.

### 2.3. Measurement Methods

After exposing and culturing, the rice seedlings were removed from the conical flasks, washed, and the root length and shoot length of each seedling were measured separately using a ruler to calculate the average values. The surface moisture of the rice seedlings was blotted dry with absorbent paper, and they were then weighed on a balance to obtain the fresh weight of each seedling. For dry biomass determination, seedlings were placed in labeled paper envelopes and dried at 80 °C to constant weight (defined as two consecutive measurements with a difference < 0.001 g). Dry weight was recorded using an analytical balance.

The POD activity in rice seedling shoots and roots was determined using a commercial POD activity assay kit (Solarbio, Beijing, China). Shoots and roots were harvested separately, and approximately 0.6 g of fresh tissue was weighed for each sample. Each tissue sample was homogenized in a mortar with 1 mL of the kit-provided POD extraction buffer. The homogenate was transferred into a centrifuge tube and centrifuged at 8000× *g* and 4 °C for 10 min. The resulting supernatant was collected and kept on ice for subsequent analysis.

Use the spectrophotometer (721G, Shanghai Yile Scientific Instrument Co., Ltd., Shanghai, China) to preheat for more than 30 min at a wavelength of 470 nm. Zero the instrument with distilled water. Measure 8.1 mL of reagent one, 3.9 mL of reagent two, and 4.05 mL of reagent three separately and add them to a conical flask. Add 8.1 mL of distilled water and mix well. Take one cuvette, use a pipette to add the above test solution 1055 μL, then add 15 μL of sample solution, and immediately turn on the stopwatch. Quickly shake evenly and place it in the spectrophotometer. Read the number once at 30 min (A1), and then read it again at 1 min and 30 s (A2). Record the data and repeat the above steps until the end. The absorbance change (∆A) is calculated as follows:

ΔA=A2−A1

The POD of rice seedlings is calculated based on sample quality, with each gram of tissue changing by 0.01 per minute in each milliliter of the reaction system as one enzyme activity unit. The calculation formula is as follows:

POD Activity = 7133×ΔA÷W

where W represents the mass of the sample, and in this experiment, W is taken as 0.6 g.

Wash the rice samples and dry them, then grind them into fine powder. Accurately weigh approximately 0.5 g of the powder and transfer it to a digestion bottle. Add 8 mL of nitric acid, and then conduct digestion in a digester at 135 °C for 5 h. Subsequently, transfer the digested solution to a test tube, add an appropriate amount of potassium persulfate for oxidation treatment, and make up the volume to 50 mL. Then, take an appropriate amount of the digested solution sample and use atomic absorption spectrometry to determine the Cu content in the sample.

### 2.4. Data Analysis

Each treatment included five independent flasks (n = 5), with three seedlings per flask. For each flask, measurements were taken for the three seedlings and averaged to obtain one value per flask. These flask means were used for statistical analyses. Data are presented as mean ± standard deviation (SD). Differences among treatments were evaluated using one-way analysis of variance (ANOVA). When significant effects were detected, Tukey’s honestly significant difference (HSD) post hoc test was applied for multiple comparisons at *p* < 0.05. Statistical analyses were performed using SPSS 27.0, and figures were prepared using Origin 2024.

## 3. Results and Discussion

### 3.1. Effects of PP-MPs and Cu on Rice Growth

**Figure 1:**
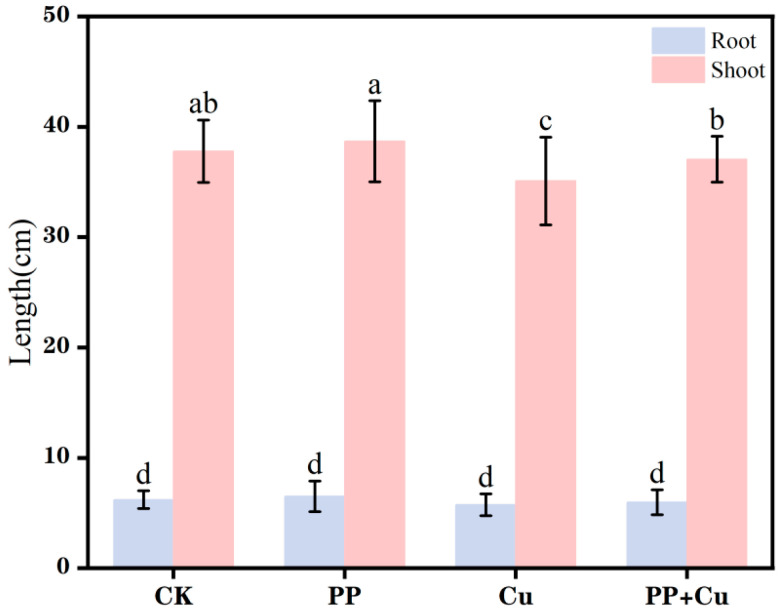
The root and shoot lengths of Rice under different treatments. Values are mean ± SD (n = 5 flasks per treatment). Different letters indicate significant differences (Tukey’s HSD, *p* < 0.05).

The root and shoot lengths of rice under different treatments are shown in Figure 1. Relative to the control group (CK), the PP treatment alone slightly promoted both root length and shoot length, with increases of 5.0% and 2.4%, respectively. The increase in root length was slightly higher than that of shoot length, which may suggest that the roots are more sensitive to microplastic stress than the aerial parts. Additionally, the growth of rice leaves in the PP treatment group did not differ markedly from that of the control group, with no signs of yellowing. However, the Cu treatment group exhibited significant yellowing of the shoots, indicating the toxicity of Cu and its damaging effects on the leaves. Cu also significantly inhibited rice growth, resulting in reduced root and shoot lengths, with the inhibition in shoots being slightly less than that in roots. This may be due to the relatively larger biomass of the shoot, which dilutes the toxic effect of Cu^2+^ [31]. Another possible reason is that the metal concentration in the roots is higher, leading to a more pronounced inhibitory effect on root length. Throughout the growth period, metals are translocated from roots to aboveground tissues, accumulating in the stems, which also inhibits the growth of the aerial parts [32].

Concomitant exposure to PP-MPs and Cu resulted in a phenotypic recovery, effectively counteracting the growth suppression typically induced by copper stress alone. Previous studies have shown that microplastics can reduce the toxicity of copper [33]. The underlying mechanism may involve the adsorption of Cu^2+^ by PP, thereby diminishing its bioavailability and mitigating copper uptake in rice plants exposed to the co-contamination scenario, thereby counteracting its direct toxic effects on rice growth. However, some leaves exhibited a certain degree of chlorosis in the combined treatment group, indicating that the alleviating effect of PP-MPs is not complete and that some Cu^2+^ still entered the plant and caused toxicity.

### 3.2. Effects of PP-MPs and Cu on Rice Biomass

**Figure 2:**
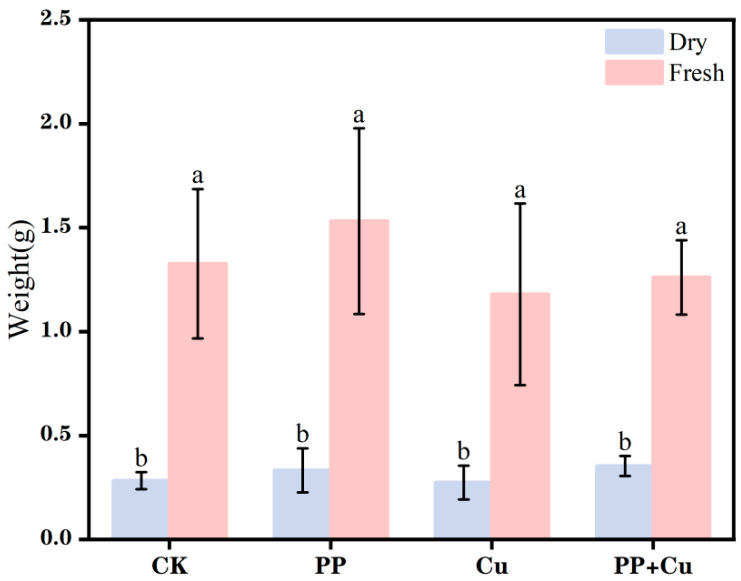
The root and shoot weights of rice under different treatments. Values are mean ± SD (n = 5 flasks per treatment). Different letters indicate significant differences (Tukey’s HSD, *p* < 0.05).

The weights of the rice roots and shoots under different treatments are shown in Figure 2. Compared to CK group, the addition of PP alone resulted in an increase in both the dry weight and fresh weight of rice seedlings. This phenomenon is consistent with the changes in root and shoot lengths, further indicating that the current concentration of PP slightly promoted rice growth. However, when Cu was added alone, the growth of the rice seedlings was inhibited in terms of both dry and fresh biomass, indicating that Cu has a negative effect on rice seedling growth and inhibits its growth.

Compared with the Cu-only treatment, the co-exposure group exhibited enhanced biomass accumulation in rice seedlings, as reflected by increases in both dry and fresh weights. This suggests that PP can partially alleviate the toxic effects of Cu^2+^, further indicating that the presence of MPs may alter the form transformation and migration of heavy metals [24].

### 3.3. Effects of PP-MPs and Cu on Peroxidase Activity in Rice

**Figure 3:**
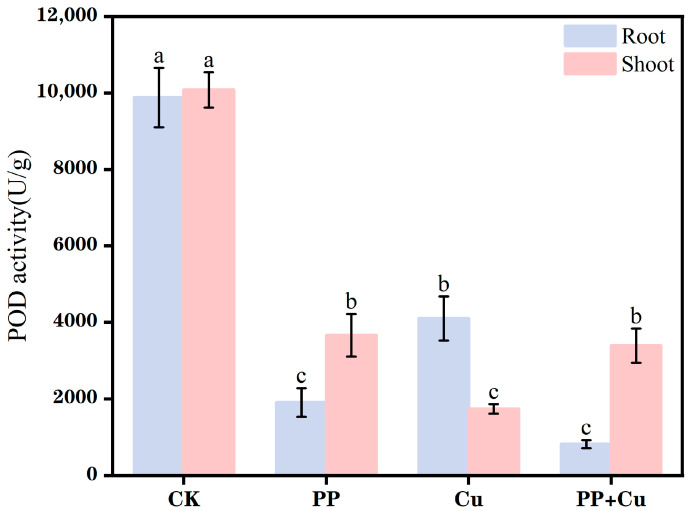
The root and shoot POD activity of rice under different treatments. Values are mean ± SD (n = 5 flasks per treatment). Different letters indicate significant differences (Tukey’s HSD, *p* < 0.05).

POD is one of the important protective enzymes in plants, which can eliminate H_2_O_2_ in cells and prevent H_2_O_2_ from attacking membrane lipids [34]. POD activity is a key component of the antioxidant defense system. This mechanism allows plants to adapt to environmental stress and maintain normal growth and development [20]. As shown in Figure 3, in the CK group, high levels of POD activity were detected in rice root and shoot parts, indicating normal growth. However, in all pollution treatment groups, the POD activity in both the roots and shoots significantly decreased (*p* < 0.05), indicating that the addition of Cu and PP negatively affected the POD activity in rice.

In the shoots, the Cu-only exposure group exhibited the most severe inhibition, with a reduction of 82.8%. In contrast, the PP + Cu combined treatment group showed a more moderate suppression of POD activity, with results similar to the PP-only exposure group. This suggests that Cu has a toxic effect on rice, while PP can alleviate the toxic effects of Cu in the shoots, supporting the conclusions from growth indicators in Section 3.1 and Section 3.2, where PP lowers the bioactivity of Cu through adsorption, thereby playing a protective role. Additionally, PP itself negatively affected POD activity in the rice shoots, which is likely due to PP attaching to the cell wall pores of the rice, causing physical obstructions [35], thereby hindering water and nutrient uptake and ultimately affecting the antioxidant response.

The phenomenon in the roots was opposite to that in the shoots. In the roots, the PP + Cu combined treatment group exhibited the most severe inhibition, with a reduction of 91.7%. The inhibition in the Cu-only exposure group was less pronounced, and the PP-only exposure group showed a suppression rate of 80.7%. This indicates that PP has a more significant inhibitory effect on POD activity in the roots, likely due to PP and the toxic Cu particles adhering to the rice roots, affecting material exchange. Compared to the shoots, the suppression effect of Cu in the roots was lower, suggesting that the addition of PP influenced the transport of active oxygen within the rice, causing a shift in its accumulation, which led to different changes in POD activity between the shoots and roots.

Although POD is commonly induced under mild or short-term stress, antioxidant enzyme activities do not necessarily increase monotonically with stress intensity. Under severe or prolonged stress, POD activity may decline due to enzyme inactivation and oxidative damage to proteins, disruption of cellular homeostasis, and suppression of enzyme synthesis caused by metabolic impairment. In the present 7-day exposure, POD activity in both shoots and roots decreased significantly in all pollutant treatments compared with CK. The antioxidant defense system was inhibited/overwhelmed at the tested stress levels. Excess Cu is known to cause strong phytotoxicity and can directly interfere with enzyme function, while PP-MPs may further constrain root surface exchange and water/nutrient uptake, indirectly limiting antioxidant enzyme production. Therefore, the higher POD activity observed in the control group represents normal physiological status, whereas reduced POD activity under treatments reflects stress-induced impairment of the antioxidant system rather than an absence of oxidative challenge.

### 3.4. Cu^2+^ Content in Rice Seedlings

**Figure 4:**
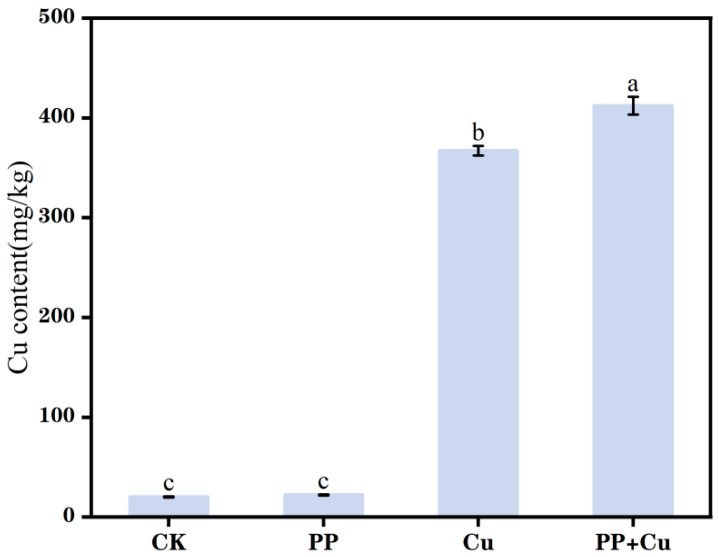
The Cu content of rice under different treatments. Values are mean ± SD (n = 5 flasks per treatment). Different letters indicate significant differences (Tukey’s HSD, *p* < 0.05).

The Cu^2+^ content in rice under different treatments is shown in Figure 4. In contrast to the CK group, both the Cu treatment group and the combined treatment group exhibited a significant elevation in Cu^2+^ content. In comparison to the Cu-only treatment, the Cu content in the rice seedlings of the combined treatment group also increased significantly, with the rice seedlings’ absorption of Cu increasing by 12.3%. Liu et al. [36] and Dong et al. [15] researched the combined stress of microplastics and cadmium on plants, found that the level of heavy metal absorption by plants is affected by the concentration and particle size of MPs, indicating that the size and concentration of MPs may be key factors leading to differences in experimental results. Based on the results of this experiment, MPs at this concentration promoted the absorption of heavy metal Cu by rice seedlings.

Copper-induced growth inhibition is commonly associated with oxidative stress and the ensuing metabolic disruption, because excess Cu can promote reactive oxygen species (ROS) production and lipid peroxidation and interfere with photosynthesis and enzyme function. Notably, mechanistic evidence from studies on copper oxide nanoparticles (CuO NPs) provides a useful reference for interpreting Cu uptake and toxicity under ionic Cu exposure [37]. CuO NP phytotoxicity can, to a large extent, be attributed to nanoparticle dissolution and the activity of released Cu^2+^ in the rhizosphere, which may subsequently drive changes in root morphology and function. Therefore, although the present study employed soluble Cu^2+^ rather than CuO NPs, these nanoparticle-based mechanisms support our interpretation that Cu-related growth suppression and antioxidant perturbations primarily reflect an oxidative stress–linked response, and they further highlight the critical role of Cu bioavailability in shaping plant outcomes.

## 4. Conclusions

This study examined the individual and combined impacts of polypropylene microplastics (PP-MPs) and copper (Cu^2+^) on rice seedling growth, peroxidase (POD) activity, and total Cu accumulation under hydroponic conditions. The primary findings are summarized as follows:

(1) PP-MPs alone slightly promoted rice seedling growth in the hydroponic system, indicating that PP-MPs can influence seedling performance in the exposure medium; however, extrapolation to soil-based systems requires further verification.

(2) Cu^2+^ exposure caused marked phytotoxicity, evidenced by leaf yellowing and reduced root and shoot growth relative to the control, confirming significant growth inhibition under the tested Cu level.

(3) Under co-exposure (PP+Cu), shoot growth performance was partially improved compared with the Cu-only treatment, and the suppression of shoot POD activity was less severe, suggesting a partial mitigation of Cu-induced toxicity at the shoot level. While this pattern is consistent with a potential reduction in effective Cu toxicity, these mechanisms were not directly quantified in this study and therefore remain to be validated.

(4) In contrast to the shoot response, the combined treatment resulted in the strongest inhibition of root POD activity (91.7% decrease) and a 12.3% increase in total Cu accumulation compared with Cu alone, indicating that PP-MPs can simultaneously enhance Cu uptake while producing divergent root–shoot antioxidant responses. Together, these findings highlight complex interactions between PP-MPs and Cu^2+^ during co-contamination.

Future studies should validate the observed PP-MP–Cu interactions in soil-based systems that better represent rhizosphere processes, and extend the exposure duration to capture longer-term responses across different microplastic types, sizes, and environmentally relevant concentration ranges.

## Data Availability

All related data are provided within the manuscript.
